# Human DIMT1 generates *N*_2_^6,6^A-dimethylation–containing small RNAs

**DOI:** 10.1016/j.jbc.2021.101146

**Published:** 2021-08-30

**Authors:** Hui Shen, Yulia Gonskikh, Julian Stoute, Kathy Fange Liu

**Affiliations:** 1 Department of Biochemistry and Biophysics, Perelman School of Medicine, University of Pennsylvania, Philadelphia, Pennsylvania, USA; 2 Graduate Group in Biochemistry and Molecular Biophysics, Perelman School of Medicine, University of Pennsylvania, Philadelphia, Pennsylvania, USA

**Keywords:** 18*S* rRNA, small RNA, DIMT1, catalytic role, RNA methylation, ribosome assembly, AML, acute myeloid leukemia, CHX, cycloheximide, DIMT1, dimethyladenosine transferase 1, HA, hemagglutinin, HEK 293T, human embryonic kidney 293T cells, HPG, homopropargylglycine, nt, nucleotide, PBST, PBS with Tween-20, poly(A)-RNA, polyadenylation RNA

## Abstract

Dimethyladenosine transferase 1 (DIMT1) is an evolutionarily conserved RNA *N*^6,6^-dimethyladenosine (m_2_^6,6^A) methyltransferase. DIMT1 plays an important role in ribosome biogenesis, and the catalytic activity of DIMT1 is indispensable for cell viability and protein synthesis. A few RNA-modifying enzymes can install the same modification in multiple RNA species. However, whether DIMT1 can work on RNA species other than 18*S* rRNA is unclear. Here, we describe that DIMT1 generates m_2_^6,6^A not only in 18*S* rRNA but also in small RNAs. In addition, m_2_^6,6^A in small RNAs were significantly decreased in cells expressing catalytically inactive DIMT1 variants (E85A or NLPY variants) compared with cells expressing wildtype DIMT1. Both E85A and NLPY DIMT1 variant cells present decreased protein synthesis and cell viability. Furthermore, we observed that DIMT1 is highly expressed in human cancers, including acute myeloid leukemia. Our data suggest that downregulation of DIMT1 in acute myeloid leukemia cells leads to a decreased m_2_^6,6^A level in small RNAs. Together, these data suggest that DIMT1 not only installs m_2_^6,6^A in 18*S* rRNA but also generates m_2_^6,6^A-containing small RNAs, both of which potentially contribute to the impact of DIMT1 on cell viability and gene expression.

DIMT1, a SAM-dependent methyltransferase installing m_2_^6,6^A in 18*S* rRNA, is conserved in all three kingdoms of life (1) (Fig. 1*A*). DIMT1 is an essential protein in a living organism (1, 2, 3, 4, 5). Elevated expression of DIMT1 is positively correlated with cell proliferation in cancer and the progression of multiple myeloma and colon cancer (6, 7, 8, 9). Despite the biological significance of DIMT1, the detailed molecular function and underlying mechanisms by which DIMT1 regulates cell viability are not fully understood, especially in humans. Disruption of the *DIMT1* gene leads to increased sensitivity of organisms to stress conditions (2, 3, 4). Interestingly, previous studies suggested that the expression, but not the catalytic activity of DIMT1, is important for rRNA processing and ribosome biogenesis (1). Bacteria with catalytically dead KsgA, which is the homolog of DIMT1 in bacteria, are more sensitive to antibiotics (10); however, in yeast, the catalytically inactive DIMT1 variants do not lead to obvious growth defects in comparison to the wildtype strain (3).

**Figure 1:**
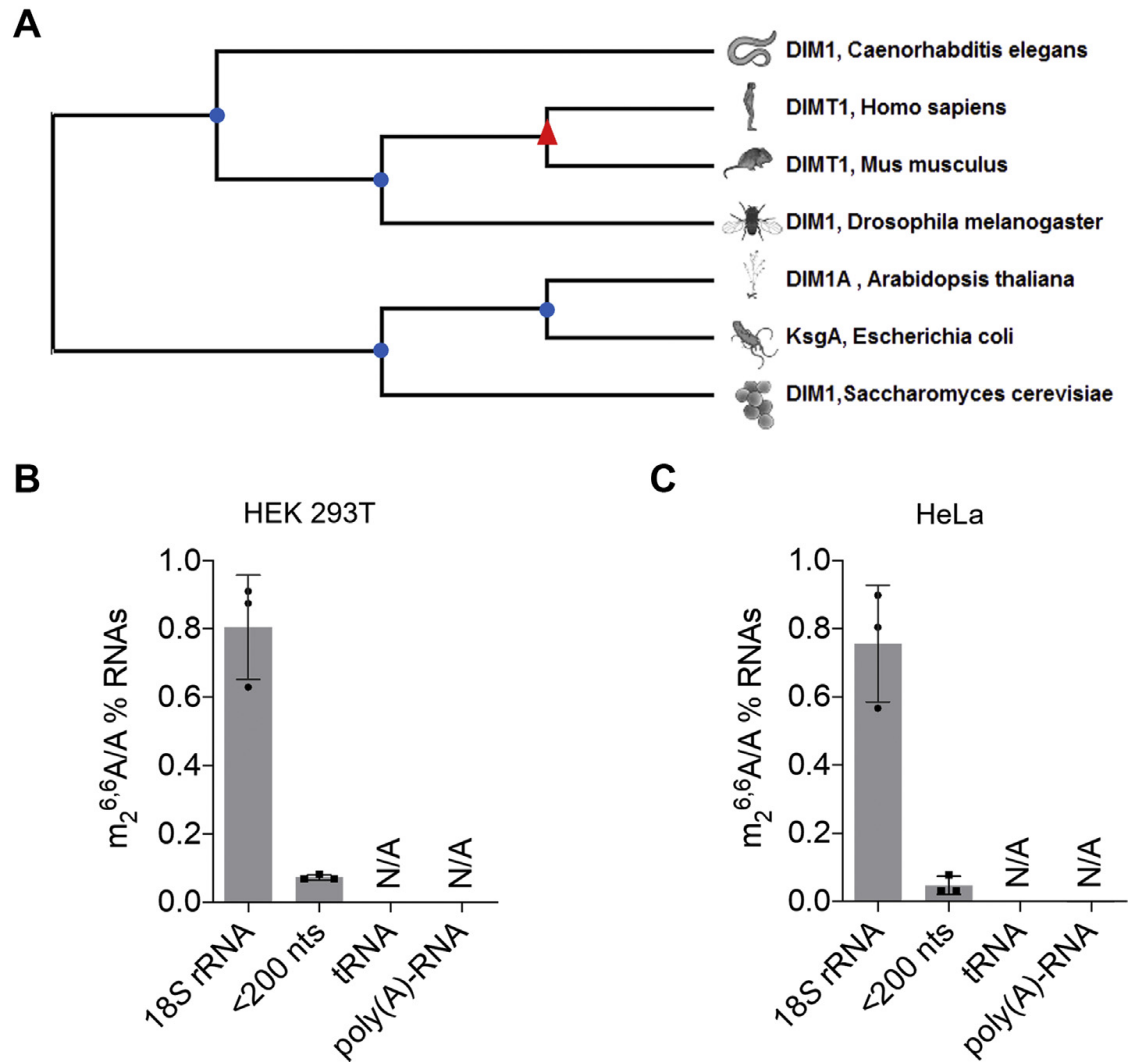
**m**_**2**_^**6,6**^**A exists in both 18*S* rRNA and small RNAs.***A*, phylogenetic analysis of DIMT1. Analysis was conducted using MEGA-X. LC–MS/MS quantification of m_2_^6,6^A in 18*S* rRNA, <200 nts RNA, tRNA, and poly(A)-RNA extracted from (*B*) HEK 293T and (*C*) HeLa cells. *p* Values were determined using a two-tailed Student's *t* test for unpaired samples. Error bars represent mean ± SD, for LC–MS/MS sample. DIMT1, dimethyladenosine transferase 1; HEK 293T, human embryonic kidney 293T cells; N/A, not determined.

In humans, m_2_^6,6^A is installed at the two adjacent adenosine sites A1850 and A1851 in 18*S* rRNA (11, 12, 13, 14, 15). The two adjacent m_2_^6,6^A are located at the interface between the 40*S* ribosome small subunit and the 60*S* large subunit, contacting tRNA and mRNA during translation. These two m_2_^6,6^A sites in 18*S* rRNA are nearly fully methylated (96% occupancy) (1, 16). Previous structural studies of the ribosomal complex suggest that DIMT1 functions as a checkpoint in translation by interacting with small subunits of the ribosome and precluding eukaryotic translation initiation factor 1A from engaging in premature translation (17, 18, 19). However, the catalytic activity of DIMT1 is not required for 18*S* rRNA processing and ribosome assembly (1), with the catalytic function of DIMT1 not fully defined. Our recent study suggests that the catalytic activity of DIMT1 is indispensable for cell viability and translation fidelity. Ribosomes with deficiency of DIMT1-mediated m_2_^6,6^A sites in 18*S* rRNA are vulnerable to programmed ribosomal frameshifting and impair internal ribosome entry site–dependent translation (5). This discovery emphasized the important functions of the rRNA dimethylation. Indeed, changes in the rRNA modification pattern have been observed in response to environmental changes during development and in disease. In human cells, the best-characterized rRNA modifications are 2′-*O*-methylation and pseudouridylation (20, 21, 22). Dysregulation of these two rRNA modifications can affect ribosome ligand binding and translation fidelity (20, 22). However, most ribosomal pseudouridines and 2′-*O*-methyl groups are dispensable for cell growth for lower eukaryotes (23, 24). The functional significance of rRNA-modifying enzymes is evidenced by the fact that dysregulation of RNA modification status or mutation in RNA-modifying enzymes has been linked to a battery of human diseases (25).

RNA modifications not only exist in rRNA but also in other RNA species, including small RNAs. There are multiple types of small RNAs such as 5*S* rRNA, snRNA, tRNAs, and microRNAs containing enzyme-mediated chemical modifications (26). Critically, these modifications on small RNAs can dramatically influence the functions of small RNAs. For instance, in the major spliceosomal snRNAs, multiple pseudouridines and 2′-*O*-methylated nucleotides have been detected (27). tRNA is the most heavily modified small RNA species; the tRNA modifications are mainly important for tRNA stability and translation fidelity (28, 29). Also, microRNAs carry modifications, such as 2′-*O*-methylation, *N*^7^-methylguanosine (m^7^G), and 8-oxoguanine2 (O^8^G), which have been shown to alter the base-pairing ability of microRNA to the mRNA targets (30, 31, 32).

Interestingly, several RNA-modifying enzymes can install the same modification in multiple types of RNAs, and the modifications are of importance for all these substrate RNAs. For instance, METTL1 installs m^7^G in both tRNA and microRNA (32, 33). m^7^G increases the stability of the modified tRNA and influences the base-pairing ability of the modified microRNAs to the mRNA targets (32, 33). Likewise, TRMT6/61 heterocomplex installs *N*^1^-methylguanosine in tRNA, which is critical for the stability of tRNA^iMet^, whereas m^1^A installation by TRMT6/61 heterocomplex in mRNA influences translation property (34, 35, 36). Here, we investigated whether DIMT1 generates m_2_^6,6^A-containing RNA species other than 18*S* rRNA and the potential biological function from DIMT1-mediated m_2_^6,6^A on RNA species.

## Results

To study whether m_2_^6,6^A exists in RNA species other than 18*S* rRNA, we first quantified the levels of m_2_^6,6^A in several major RNA species (Fig. 1*B*). We extracted 18*S* rRNA, tRNA, and RNA species with a size smaller than 200 nucleotides (nts) using size fractionation from two mammalian cell lines (Fig. S1, *A* and *B*). In addition, we extracted polyadenylation RNA (poly(A)-RNA) using two rounds of poly-dT extraction followed by one round of RiboMinus purification (Fig. S1*C*). We performed quantitative RT-PCR to quantify the levels of 18*S* rRNA in the samples from each of poly(A)-RNA purification steps. The results showed that two rounds of polydT extraction followed by one round of RiboMinus purification lead to inappreciable levels of 18*S* rRNA in the remaining RNA species, which is also supported by the bioanalyzer results (Fig. S1*C*). The extracted RNAs were then digested to single nucleosides before being analyzed *via* triple quadrupole LC–MS/MS. The LC–MS/MS results showed that, among these different RNA species, m_2_^6,6^A is more significantly enriched in, in addition to 18*S* RNA, RNA species with a size smaller than 200 nts in both HeLa and human embryonic kidney 293T (HEK 293T) cell lines (Fig. 1, *B* and *C*, Figs. S1*D*, and S2).

**Figure 2:**
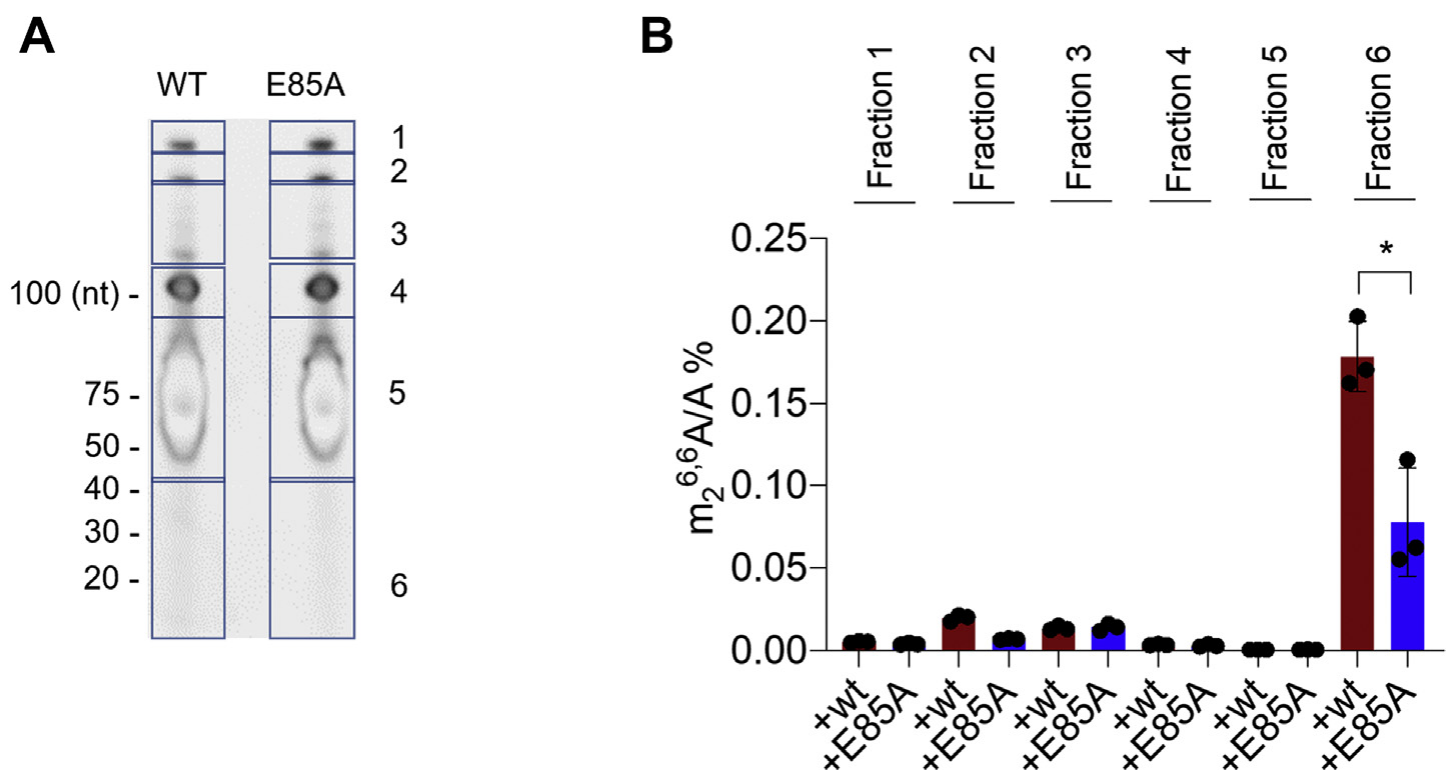
**DIMT1 catalytic inactive variants lead to decreased m**_**2**_^**6,6**^**A levels in small RNAs (<40 nts).***A*, TBE–urea gel purification of major fractions (1–6 as shown in the figure) in small RNAs extracted from wildtype and E85A DIMT1 HEK 293T cells. Band #1: U2 snRNA; #2: U1 and 5.8*S* rRNA; #3: U4 snRNA; #4: 5*S* rRNA, U5, U6 snRNAs; #5: tRNA; and #6: small RNAs including microRNAs and RNA fragments. LC–MS/MS quantification of (*B*) RNAs extracted from bands 1 to 6 in TBE–urea gel purification from (*A*). DIMT1, dimethyladenosine transferase 1; HEK 293T, human embryonic kidney 293T cells.

To study whether DIMT1 controls m_2_^6,6^A levels in the small RNAs (<200 nts) through catalysis, we employed a *DIMT1*^+/−^ heterozygous cell line, which we established previously (5). We further performed reconstitution of empty vector, wildtype, and E85A DIMT1, respectively, in this *DIMT1*^+/−^ heterozygous cell line (5). As shown in Fig. S3, *A* and *B*, FLAG-tagged exogenous wildtype and FLAG-tagged E85A DIMT1 expressed at the comparable levels in *DIMT1*^+/−^ heterozygous cells; the remaining endogenous DIMT1 also expressed at the comparable levels in these cells. Next, the small RNAs (<200 nts) were first extracted and further separated by denaturing urea polyacrylamide gel (37, 38). As shown in Figure 2*A*, we extracted the major small RNA species based on their sizes and subjected each fraction to LC–MS/MS analysis. The major types of small RNAs in each size fraction were cut and extracted from the gel (Fig. 2*A*). RNAs with a size smaller than 40 nts were strikingly enriched in m_2_^6,6^A, whereas other RNAs in fractions 1 to 5 had extremely low m_2_^6,6^A, which are mainly splicesome RNA and tRNA (Fig. 2*B*). Expression of the catalytically inactive DIMT1 variant E85A leads to a decreased m_2_^6,6^A level in RNA below 40 nts in comparison to wildtype DIMT1 (Fig. 2*B* and Fig. S3*C*). Likewise, *DIMT1*^+/−^ cells have a significantly lower level of m_2_^6,6^A in RNA below 40 nts than wildtype HEK cells (Fig. S4).

**Figure 3:**
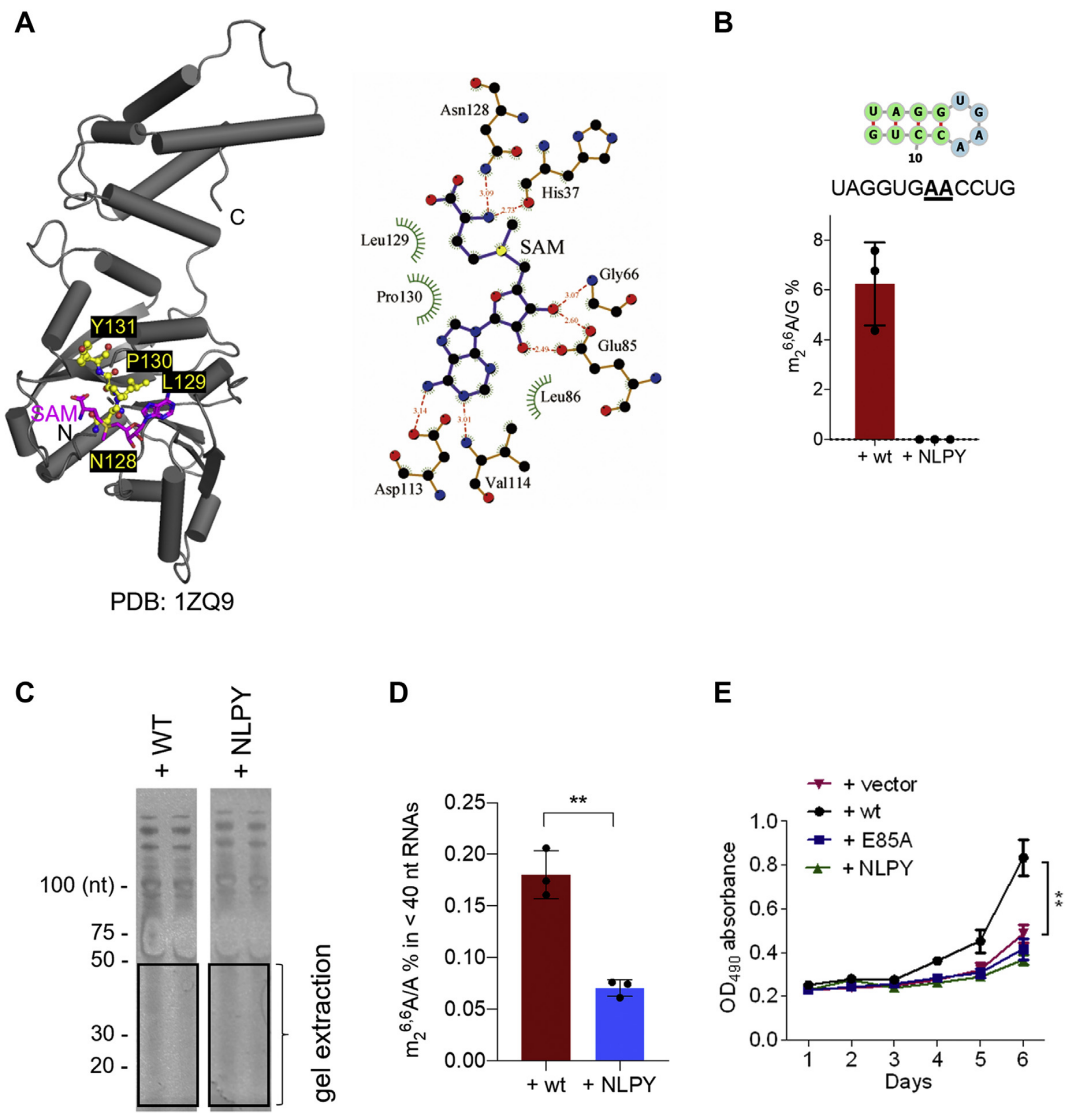
**Identification of NLPY DIMT1 as catalytically inactive.***A*, crystal structure of wildtype DIMT1 (Protein Data Bank: 1ZQ9). Four amino acids (Asn128Leu129Pro130Tyr131) were shown directly contacting SAM. *B*, LC–MS/MS quantification of m_2_^6,6^A levels in an unmodified RNA probe after *in vitro* methylation reaction with wildtype or NLPY DIMT1 variant. SAM was supplemented in all the *in vitro* methylation activity assays. *C*, TBE–urea gel purification of small RNAs extracted from wildtype and NPLY DIMT1 HEK 293T cells. *D*, LC–MS/MS quantification of RNAs extracted from the annotated region in (*C*). *E*, cell proliferation assays performed in *DIMT1*^+/−^ + empty vector, *DIMT1*^+/−^ + wildtype (wt), *DIMT1*^+/−^ + E85A, and *DIMT1*^+/−^ + NLPY cells. *p* Values were determined using a two-tailed Student's *t* test for unpaired samples. Error bars represent mean ± SD. ∗∗*p* < 0.01. DIMT1, dimethyladenosine transferase 1; HEK 293T, human embryonic kidney 293T cells.

To further investigate whether the catalytic role of DIMT1 is required for the generation of m_2_^6,6^A-containing small RNAs, we mutated four residues (amino acids 128–131 Asn, Leu, Pro, and Tyr) that directly bind to SAM to generate the NLPY128 to 131AAAA DIMT1 variant (referred to as NLPY hereafter) (Fig. 3*A*). We expressed and purified full-length recombinant wildtype and NLPY DIMT1 proteins (Fig. S5*A*). The purified NLPY DIMT1 variant has similar folding and stability property as the recombinant wildtype DIMT1 (Fig. S5*B*). We then performed *in vitro* methylation assays using the DIMT1 NLPY variant and wildtype DIMT1 with a synthetic RNA probe bearing the same local structure as the two m_2_^6,6^A sites in the 18*S* rRNA 45 helix (Fig. 3*B*). The LC–MS/MS results showed that wildtype DIMT1 effectively installs m_2_^6,6^A in this RNA probe; in contrast, the NLPY DIMT1 variant failed to install m_2_^6,6^A level in the RNA probe (Fig. 3*B* and Fig. S5, *C* and *D*). To measure the methyltransferase activity of the NLPY DIMT1 variant on small RNAs inside cells, we constructed cell lines stably expressing either FLAG-tagged NLPY in our previously established *DIMT1*^+/−^ HEK 293T cells (Fig. S3). Similarly, we purified small RNAs by sizes using wildtype DIMT1 and NLPY mutant cells. The results showed that NLPY DIMT1, which is consistently observed with E85A DIMT1, leads to an obvious decrease of m_2_^6,6^A in RNA below 40 nts in comparison to wildtype DIMT1 (Fig. 3*D* and Fig. S5, *E* and *F*). In mammalian cells, RNAs smaller than 40 nts are mainly miRNAs, tRNA fragments, and rRNA fragments. Since unfragmented tRNAs do not have m_2_^6,6^A, it is less likely that m_2_^6,6^A in <40 nts RNAs comes from tRNA fragments. Like E85A DIMT1 variant cells, NLPY variant cells also present significantly impaired cell proliferation compared with the wildtype cells, which suggests that the catalytic activity of DIMT1 promotes cell viability (Fig. 3*E*).

Since we observed that the E85A DIMT1 variant also leads to a significant impact on m_2_^6,6^A levels in 18*S* rRNA and protein synthesis (5), we studied the impact of NLPY DIMT1 variant on the level of m_2_^6,6^A on 18*S* rRNA, 18*S* processing, 40*S* assembly, and global protein synthesis. As shown in Fig. S6*A*, LC–MS/MS results suggest that the cells expressing the NLPY variant have a greatly decreased level of m_2_^6,6^A in 18*S* rRNA compared with the cells expressing wildtype DIMT1, which is consistent with the E85A DIMT1 variant result (5). In addition, the NLPY DIMT1 variant did not lead to noticeable changes in the ratio of 18*S* to 28*S* rRNA (Fig. S1*B*). The results from quantitative RT-PCR quantification of 18*S* rRNA extracted from wildtype and NLPY DIMT1 variants are consistent with the results from the agarose gel images (Fig. S6*B*). To analyze whether the NLPY DIMT1 variant impairs 40*S* assembly, we conducted polysome profiling using cell lysate from *DIMT1*^+/−^ + wildtype and *DIMT1*^+/−^ + NLPY variant cell lines. As shown in Fig. S6*C*, *DIMT1*^+/−^ + NLPY variant cells do not show obvious changes in the polysome profiles compared with the *DIMT1*^+/−^ + wildtype cells. These results are consistent with our previously observed one in E85A-reconstituted *DIMT1*^+/−^ cells that the protein scaffold but not the catalytic role of DIMT1 is required for ribosome assembly (5). However, *DIMT1*^+/−^ + NLPY variant cells present decreased protein synthesis, as revealed in the homopropargylglycine (HPG) assays (Fig. S6*D*). The HPG results obtained using NLPY variant DIMT1 compared with the wildtype DIMT1 cells suggest that the catalytic role of DIMT1, although not required for 40*S* assembly, is important for protein translation.

**Figure 4:**
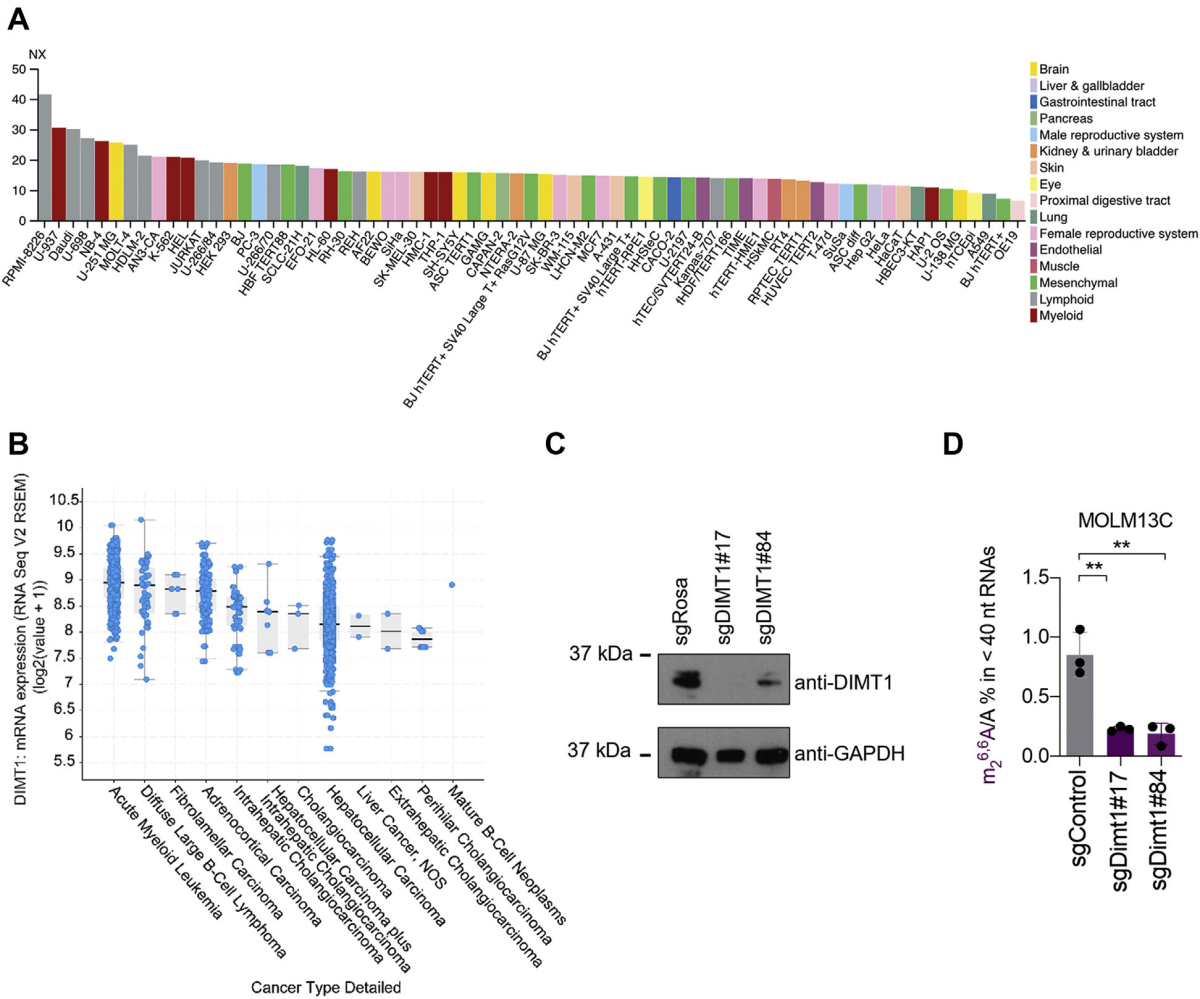
**DIMT1 is highly expressed in AML cells, and downregulation of DIMT1 leads to decreased m**_**2**_^**6,6**^**A in small RNA in AML cells.***A*, DIMT1 is highly expressed in lymphoid and myeloid cancer cells (data were obtained from the Human Protein Atlas, http://www.proteinatlas.org). *B*, high-level expression of DIMT1 in AML cells (data were obtained from The Cancer Genome Atlas). *C*, Western blots showing the expression of DIMT1 in MOLM13C cells transfected with either a control guide RNA (sgRosa) or guide RNAs targeting DIMT1 (#17 and #84). *D*, LC–MS/MS quantification of m_2_^6,6^A in small RNAs extracted from MOLM13C cells infected with either guide RNA control or guide RNA targeting DIMT1. *p* Values were determined using a two-tailed Student's *t* test for unpaired samples. Error bars represent mean ± SD; ∗∗*p* < 0.01. AML, acute myeloid leukemia; DIMT1, dimethyladenosine transferase 1.

It was previously shown that elevated expression of DIMT1 is frequently seen in human cancers (6, 7, 8, 9). Thus, we investigated if the ectopic expression of DIMT1 in cancer cells generates m_2_^6,6^A in <40 nts RNAs. We first investigated the expression of DIMT1 in different cancer samples from the Human Protein Atlas database (http://www.proteinatlas.org). As shown in Figure 4*A*, DIMT1 is highly expressed in lymphoid and myeloid cancers. Notably, DIMT1 is highly expressed in acute myeloid leukemia (AML) suggested by the data from The Cancer Genome Atlas database (Fig. 4*B*). We further quantified the levels of m_2_^6,6^A in <40 nts RNAs in MOLM-13C AML cells. The LC–MS/MS results suggest that m_2_^6,6^A naturally exists in <40 nts RNAs in MOLM-13C AML cells, and the level of which is much higher than in wildtype HEK 293T cells. Next, we employed single-guide RNA and CRISPR/Cas9 system to remove DIMT1 in the MOLM-13C AML cells. A negative control guide RNA (sgRosa) and two guide RNAs targeting DIMT1 (sgDIMT1#17 and sgDIMT1#84) were packed in lentivirus and used to infect the MOLM-13C AML cells, which stably express Cas9. We collect the cells at day 5 after viral infection. The Western blots showed that the transfection of sgDIMT1 (but not sgRosa) led to a significantly decreased protein level of DIMT1 (Fig. 4*C*). The LC–MS/MS results suggest that knockdown of DIMT1 led to a significant decrease of m_2_^6,6^A in <40 nts RNAs in MOLM-13 AML cells (Fig. 4*D* and Fig. S7).

## Discussion

While the noncatalytic functions of DIMT1 in 18*S* processing and ribosome assembly are intensively studied, the role of DIMT1-mediated m_2_^6,6^A in RNA remains elusive. In a previous study, we revealed that DIMT1-mediated m_2_^6,6^A in 18*S* rRNA is important for translational fidelity and prevents −1 ribosome frameshift (5). In this work, we show that, in addition to 18*S* rRNA, m_2_^6,6^A exists in RNA with a size smaller than 40 nts at a higher abundance compared with other major RNA species, including poly(A)-RNA, tRNA, and splicesome RNA. We further show that the lack of such a catalytic activity of human DIMT1 significantly decreases m_2_^6,6^A in both 18*S* rRNA and RNA with a size smaller than 40 nts. These data suggest that DIMT1 installs m_2_^6,6^A not only in 18*S* rRNA but possibly also generates m_2_^6,6^A-containing small RNAs in mammalian cells. Thus, the regulatory function of DIMT1 is possibly beyond the 18*S* rRNA level and should be further investigated in the future.

Most RNA species with a size smaller than 40 nts are tRNA fragments, rRNA fragments, and microRNAs, all of which have been shown to be of biological significance. Our results showed that the mature tRNAs do not have appreciable levels of m_2_^6,6^A (Fig. 1, *B* and *C*). Therefore, it is less likely that the cleavage of mature tRNA directly leads to the m_2_^6,6^A observed in the small RNA species. However, we do not exclude the possibility that DIMT1 could utilize tRNA fragments as substrates to install m_2_^6,6^A. rRNA fragments from 18*S* rRNA possibly contribute to the detected m_2_^6,6^A in small RNA species. Previous studies showed that 18*S* rRNA produces rRNA fragments of all lengths but prevalently in the sizes between 18 and 23 nts (39); thus, it is possible that the m_2_^6,6^A in small RNA species is 18*S* rRNA fragments. However, our results showed that overexpression of wildtype DIMT1 did not lead to an appreciable increase of m_2_^6,6^A in 18*S* rRNA but induced a significantly increased level of m_2_^6,6^A in small RNAs. Since m_2_^6,6^A sites are almost 100% occupant in 18*S* rRNA, it is not surprising that expressing wildtype DIMT1 cannot further increase the level of m_2_^6,6^A in 18*S* rRNA. However, the significant increase of m_2_^6,6^A in small RNAs suggests that overexpression of DIMT1 possibly installs m_2_^6,6^A in a previously unmodified site. rRNA fragments have been shown to influence gene expression through alternating decoding of genetic information (40). Thus, DIMT1 might influence gene expression through enzymatically regulating modification in rRNA fragments.

In addition to 18*S* rRNA fragments, it is also possible that DIMT1 performs *de novo* methylation on other small RNA species such as microRNAs, the primary role of which is in gene silencing and post-transcriptional gene regulation through base pairing with its mRNA targets (41). Dysregulation of microRNAs targeting is frequently seen in human disorders (42, 43, 44). Previous studies suggest that most miRNA–mRNA interactions involve the seed region at the 5′ end of the microRNA. The central region may also mediate the accurate interaction with the mRNA targets (45). Of note, m_2_^6,6^A may interfere base pairing of microRNA to its mRNA targets since m_2_^6,6^A cannot base pair with thymidine. Unfortunately, because of the technical difficulties, mainly because m_2_^6,6^A blocks reverse transcription and the already short length of small RNAs (<40 nts), we failed to reveal the identity of the small RNAs successfully.

Ectopic expression of DIMT1 has been implicated in human cancer such as AML. AML is a blood cancer characterized by uncontrolled self-renewal and blocked differentiation of myeloid cells. Many driver oncogenes in AML hijack gene regulatory pathways, developing an essential dependency on proteins governing these AML-specific programs to maintain the protumorigenic state. RNA modifications are suggested as an important regulator in AML in support of oncogenic pathways (46, 47, 48). It will certainly be interesting to study the identity and biological functions of m_2_^6,6^A-containing small RNAs in human cancers such as AML in the future.

**Figure 5:**
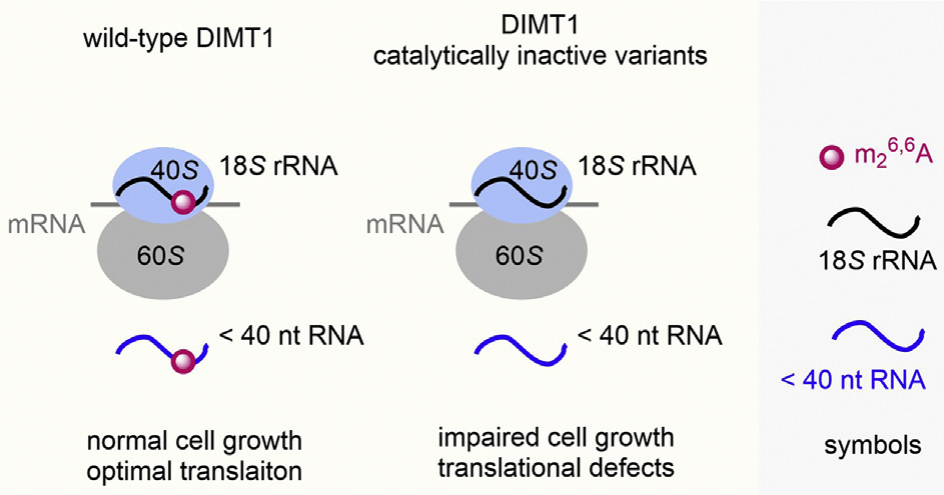
**A schematic summary of DIMT1-mediated m**_**2**_^**6,6**^**A in 18*S* rRNA and small RNAs. DIMT1, dimethyladenosine transferase 1**.

In summary, this study continues to uncover the catalytic role of DIMT1, that is, installing m_2_^6,6^A in 18*S* rRNA and small RNA (<40 nts), and the biological consequences of lack of the catalytic role of DIMT1 in the regulation of translation and cell viability (Fig. 5). These findings provide a starting point for future investigations into the effects of DIMT1-mediated dimethylation in multiple RNA species, the mechanism by which this methylation modulates cell viability, and, more generally, expanding our view of the regulatory controls exerted by DIMT1 across rRNA and small RNAs.

## Experimental procedures

### Construction, expression, and purification

The Human *DIMT1* gene (Gene ID: 27292) was cloned from the human complementary DNA library and ligated into a pET-28a vector for protein expression and pPB vector for overexpression in mammalian cells as previously described (5).

Briefly, pET-28a plasmids were transformed into *Escherichia coli* BL21 (DE3) for purification. Expression of all recombinant proteins was induced with 0.5 mM IPTG when the cell density reached an absorbance at 600 nm between 0.6 and 0.8. After growth for approximately 20 h at 16 °C, the cells were collected and lysed in the lysis buffer (25 mM Tris, pH 7.5, and 500 mM NaCl) by sonication. After centrifugation at 4 °C for 30 min, the supernatant was loaded onto a Ni^2+^-affinity chromatography column (GE Healthcare), which was pre-equilibrated using the lysis buffer. Then, the column was washed three times with wash buffer (50 mM imidazole in lysis buffer), and the bound target protein was eluted with elution buffer (500 mM imidazole in lysis buffer). The eluted proteins were further purified using HiTrap SPFF (GE Healthcare) column using a gradient elution formed by low salt buffer (25 mM Tris, pH 7.5, and 50 mM NaCl) and high salt buffer (25 mM Tris, pH 7.5, and 1 M NaCl), and then HiLoad Superdex 200 (GE Healthcare) column with S200 buffer (25 mM Tris, pH 7.5, and 200 mM NaCl).

### *In vitro* methylation assay

The *in vitro* methylation assays were performed in a 30-μl reaction mixture containing the following components: 4 μg biotinylated RNA probe, 24 μg protein (wildtype DIMT1 or NLPY mutant), 1 mM SAM, 50 mM Tris, pH 7.5, 5 mM MgCl_2_, and 1 mM DTT. The reaction was incubated at 16 °C overnight. After incubation, streptavidin beads (Thermo Fisher Scientific) were used to purify the RNA probe, following the instruction, and eluted with RNase-free water at 75 °C for 5 min. Then, the purified RNA probe was digested and dephosphorylated to single nucleosides using Nucleoside Digestion Mix (NEB; M0649S) for LC–MS/MS quantification. The RNA probe was synthesized from IDT, and the sequence is as follows: probe 1: 5′-BiotinUAGGUGAACCUG-3′.

### Mammalian cell culture, siRNA knockdown, and plasmid transfection

HEK 293T cells were cultured in Dulbecco's modified Eagle's medium (GIBCO; 11995065) supplemented with 10% fetal bovine serum (GIBCO; 26140-079) and 1% penicillin/streptomycin (Corning; 30-002-CI) in 37 °C, 5% CO_2_ incubator. Lipofectamine 2000 (11668019) from Invitrogen was used for transfection of plasmids, following the manufacturer's instructions.

### Quantitative analysis of the m_2_^6,6^A level using LC–MS/MS

The cells were collected with a cell lifter, and the 18*S* RNA was extracted as indicated in the RNA isolation section later. Purified RNA or RNA probes were digested and dephosphorylated to single nucleosides using Nucleoside Digestion Mix (NEB; M0649S) at 37 °C for 1 h. The detailed procedure was as previously described (5). The nucleosides were quantified using retention time and the nucleoside-to-base ion mass transitions of 268.0 → 136.0 (A), 284.0 → 152.0 (G), and 296.0 → 164.1 (m_2_^6,6^A). All quantifications were performed by converting the peak area from the LC–MS/MS to moles using the standard curve obtained from pure nucleoside standards. Then, the percentage ratio of m_2_^6,6^A to A or G was used to compare the different modification levels.

### Construction of stable overexpression of cell lines

*DIMT1*^+/−^ heterozygous HEK 293T cells and FLAG-hemagglutinin (HA)–tagged wildtype DIMT1, FLAG-HA-tagged E85A mutant, or empty overexpression vector (pPB backbone)–expressing *DIMT1*^+/−^ heterozygous HEK 293T cells were constructed previously (5). Using the same method, we constructed a FLAG-HA-tagged NLPY DIMT1 stable cell line in this study. The stable overexpression was confirmed by Western blot using an anti-FLAG antibody (Thermo; MA1-91878-HRP).

### Polysome profiling

Cells were seeded in 10-cm plate 1 day before at 70% confluence. Before collecting cells, cycloheximide (CHX) was added to the cell culture media at 100 μg/ml for 7 min. Then, the media were discarded, and the cells were washed once with ice-cold 1× PBS containing CHX (100 μg/ml). The cells were collected by a cell lifter with 5-ml cold PBS containing CHX (100 μg/ml). Cells were pelleted at 500*g* for 3 min at 4 °C and resuspended with 500 μl lysis buffer (10 mM Tris, pH 7.4, 150 mM KCl, 5 mM MgCl_2_, 100 μg/ml CHX, 0.5% Triton-X-100, freshly add protease inhibitor, and 40 U/ml SUPERasin). After lysing on ice for 15 min, the supernatant was collected by centrifugation at 15,000*g* for 15 min. The cell lysate was then layered on top of a linear 10% to 50% sucrose gradient (10 mM Tris, pH 7.4, 150 mM KCl, 5 mM MgCl_2_, 100 μg/ml CHX, and 40 U/ml SUPERasin) and centrifuged at 4 °C for 150 min at 35,000 rpm (Beckman; rotor SW-40Ti). The samples were then fractioned and analyzed by Gradient Station (BioCamp) equipped with TRIAX flow cell (BioCamp).

### HPG assay

The HPG assay was performed using the Click-iT HPG Alexa Fluor 594 Protein Synthesis Assay Kits (Life Technologies; C10429), following the manufacturer's instruction. Briefly, cells were cultured in 6-well plates, with one coverslip on in each well. On the day of the experiment, the regular cell culture medium was replaced by 1 ml of l-methionine-free RPMI1640 medium containing 1 μl Click-iT reagent for 45 min. Then, the cells were washed once with 1× PBS and fixed with 4% formaldehyde in 1× PBS at room temperature for 15 min. After washing twice with 3% bovine serum albumin, the cells were permeabilized using 0.5% Triton X-100 at room temperature for 20 min. The Click-iT reaction was carried out at room temperature for 30 min followed by a quenching step. Then, the DNA was stained by HCS NuclearMask Blue Stain reagent for 3 min. Finally, the coverslips were mounted with antifade reagent (Invitrogen; P36970), and the images were captured using a Leica DM6000 motorized upright microscope with the same settings for all the images. For quantification, four cells were unbiasedly selected in each HPG figure to intensity quantification of the integrated area using Fiji. The regions next to the cells without fluorescence were similarly selected and quantified as the background. Then the following formula was used to calculate the corrected cell fluorescence intensity: integrated intensity − (area of selected cell × mean integrated intensity of backgrounds). Finally, the fluorescence intensities of *DIMT1*^+/−^ + wildtype were normalized to *DIMT1*^+/−^ + NLPY cells.

### Cell proliferation assay

The cells were first trypsinized and counted using a cell counting chamber slide (Invitrogen; 100078809). Then, 1000 cells were seeded into every single well in a 96-well plate in 100-μl cell culture medium. The next day, 20 μl of CellTiter 96 AQueous One Solution (Promega) was added to each well at 37 °C and 5% CO_2_ for 2 h. Absorbance at 490 nm was then measured using GloMax plate reader (Promega).

### Protein quantitation and Western blot

Protein concentration for samples was calculated using the Bradford Assay (catalog no.: 5000006; Bio-Rad). Protein samples were boiled at 95 °C with Laemmli sample buffer for 10 min. After brief centrifugation, samples were loaded onto SDS-PAGE gels. After running at 180 V for 1 h, the gel was transferred to polyvinylidene fluoride membranes by semidry transfer apparatus at 20 V for 50 min. Then, the polyvinylidene fluoride membranes were blocked with 5% milk in 1× PBS with Tween-20 (PBST) for 30 min at room temperature and incubated with 3% milk in 1× PBST containing corresponded antibody overnight at 4 °C. After washing three times with 1× PBST, horseradish peroxidase–conjugated secondary antibody (1:20,000) in 1% of milk was applied and incubated at room temperature for 1 h. After washing three times with 1× PBST, the membrane was visualized using ECL Western Blotting Detection Kit (Thermo Fisher Scientific).

### RNA isolation

For total RNA extraction, TRIzol reagent (Invitrogen) was used following the manufacturer's instruction.

#### 18*S* rRNA

About 5 μg of total RNA was subjected to further separation on a 1.5% agarose gel. 18*S* rRNAs in the gel slices were then extracted using Zymoclean Gel RNA Recovery kit (R1011).

#### Poly(A)-RNA isolation

mRNA was extracted from the total RNA by using Dynabeads mRNA Purification Kit (Ambion) following the instructions. The rRNA was further removed by RiboMinus Eukaryote Kit (Invitrogen). The poly(A)-RNA integrity was tested by Bioanalyzer with RNA nano Chips (Agilent Technologies).

#### Small RNA isolation

The procedure was adapted from a previous report (49). Briefly, RNA species smaller than 200 nt were extracted from the total RNA using RNA Clean & Concentrator Kits (Zymo Research). The tRNA fraction was further extracted from the small RNAs by using 6% TBE–urea gel and extracting the migrated tRNAs from the gel using a ZR small-RNA PAGE Recovery Kit (Zymo Research).

### Thermostability assay

Purified wildtype DIMT1 and NLPY mutant proteins were diluted to 0.25 mg/ml. About 19 μl of each protein was transferred to a well in a 394-well plate, and 1 μl of fivefold SYPRO Orange (Thermo Fisher Scientific; S6650) was added to each well. The plate was sealed and spun at 3600*g* for 2 min. The fluorescent signal at 570 nm was collected using an RT-qPCR machine with the temperature ramping from 20 to 95 °C. The data were analyzed using DSF World (50).

### Lentivirus package and infection

Control guide RNA and guide RNAs targeting DIMT1 were cloned into LRG 2.1T vector. About 5 μg LRG 2.1T, 2.5 μg VSVG, and 3.75 μg pPAX2 plasmids were cotransfected to HEK 293T cells in 6-well plate with 40 μl polyethylenimine. The medium was collected in a total 3-day period and spun down at 3000 rpm for 5 min at room temperature. The lentivirus was used to infect the MOLM13C cells with 8 μg/ml polybrene. The infected cells were collected at day 5 after infection for further analysis.

## Data availability

This study does not have sequencing data or structure data for deposition.

## Supporting information

This article contains supporting information.

## Conflict of interest

The authors declare that they have no conflicts of interest with the contents of this article.
