# Supplementary material for: Human DIMT1 generates N26,6A-dimethylation–containing small RNAs
Source: J Biol Chem. 2021 Aug 30;297(4):101146. doi: 10.1016/j.jbc.2021.101146 (PMC8463865; doi:10.1016/j.jbc.2021.101146)
Supplement: Supplemental Figures S1–S7 [file mmc1.pdf]

Human DIMT1 generates $N_2^{6,6}$A-dimethylation-containing small RNAs

Hui Shen[1], Yulia Gonskikh[1], Julian Stoute[1,2], Kathy Fange Liu[1,2]*

Affiliations:

[1]Department of Biochemistry and Biophysics, Perelman School of Medicine, University of Pennsylvania, Philadelphia, PA 19104, USA

[2]Graduate Group in Biochemistry and Molecular Biophysics, Perelman School of Medicine, University of Pennsylvania, Philadelphia, PA 19104, USA

*Correspondence to liufg@pennmedicine.upenn.edu

Running title: Small RNAs identified carrying $N_2^{6,6}$A-dimethylation

Keywords: 18*S* rRNA, small RNA, DIMT1, catalytic role, RNA methylation, ribosome assembly

Supporting Information

Included Supporting Information: Supplementary Figures S1-S7.

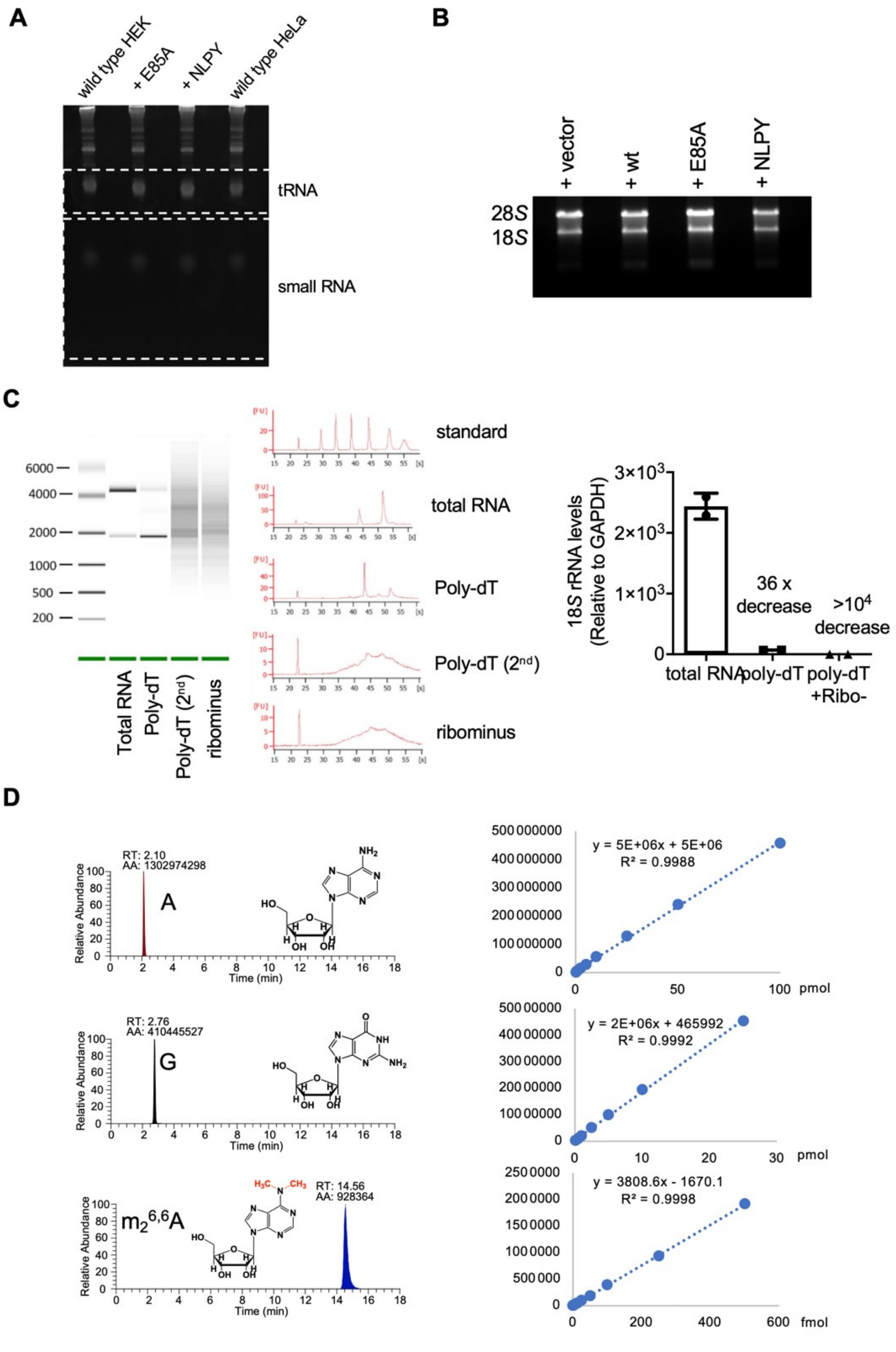

Figure S1. Purification of multiple RNA species. Purification of (A) tRNAs and (B) 18*S* and 28*S* rRNA. (C) RNA bioanalyzer results of the purification process of polyadenylated RNAs. This gel-like image is the simulated image of the transfer of the raw migration time and intensity data to mimic electrophoretic assays. qRT-PCR of 18*S* rRNA from total RNA, after two rounds of poly-dT extracted, and after one more round of ribominus purification. (D) LC-MS/MS channels, peak areas, and standard curves of adenosine, guanosine, and $m_2^{6,6}A$.

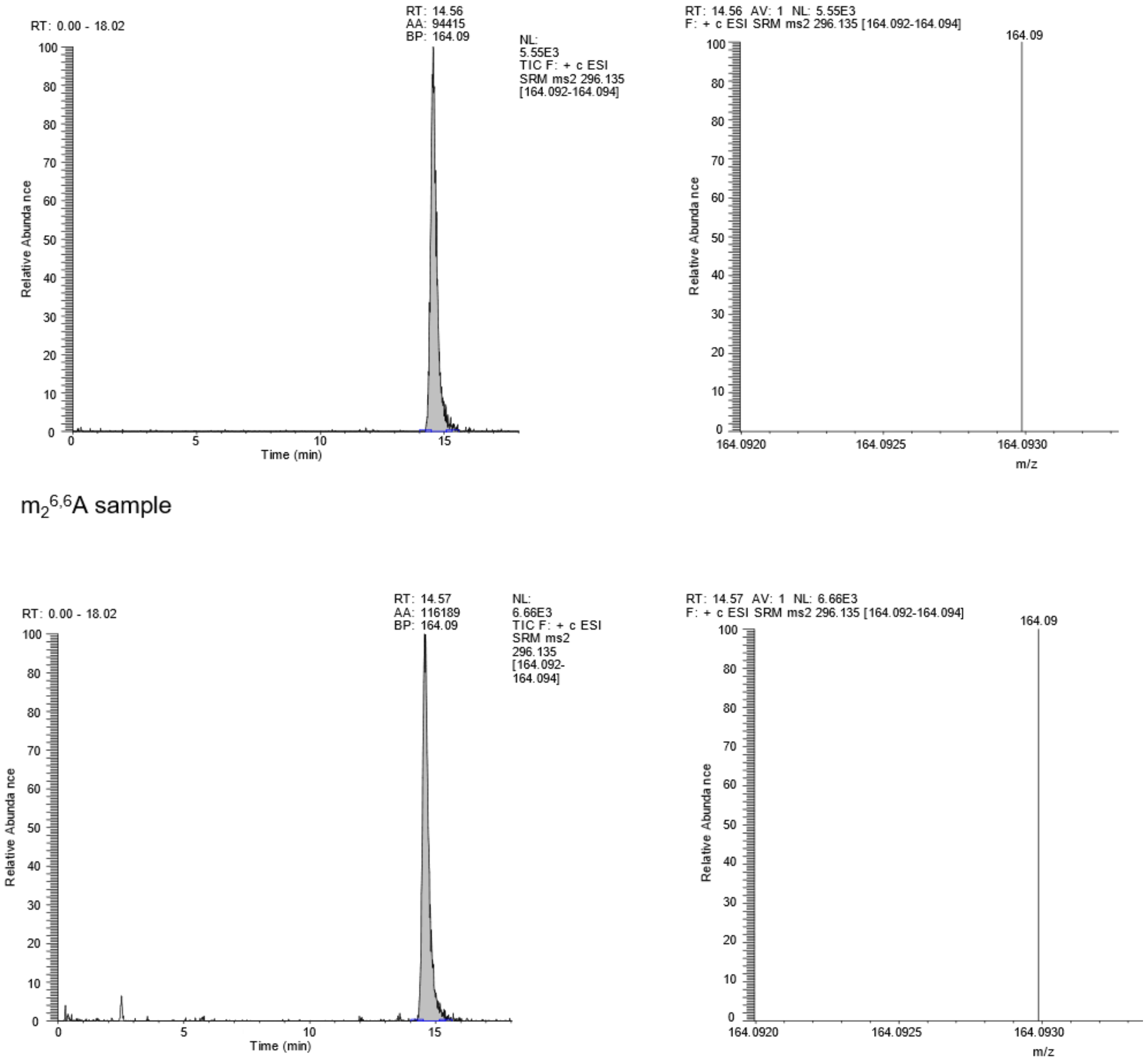

Figure S2. ESI-CID data for $m_2{}^{6,6}A$ standard (paired with chromatogram peaks), measured using (A) synthetic standards (upper panels) and 18*S* rRNA isolated from HEK 293T cells (lower panels).

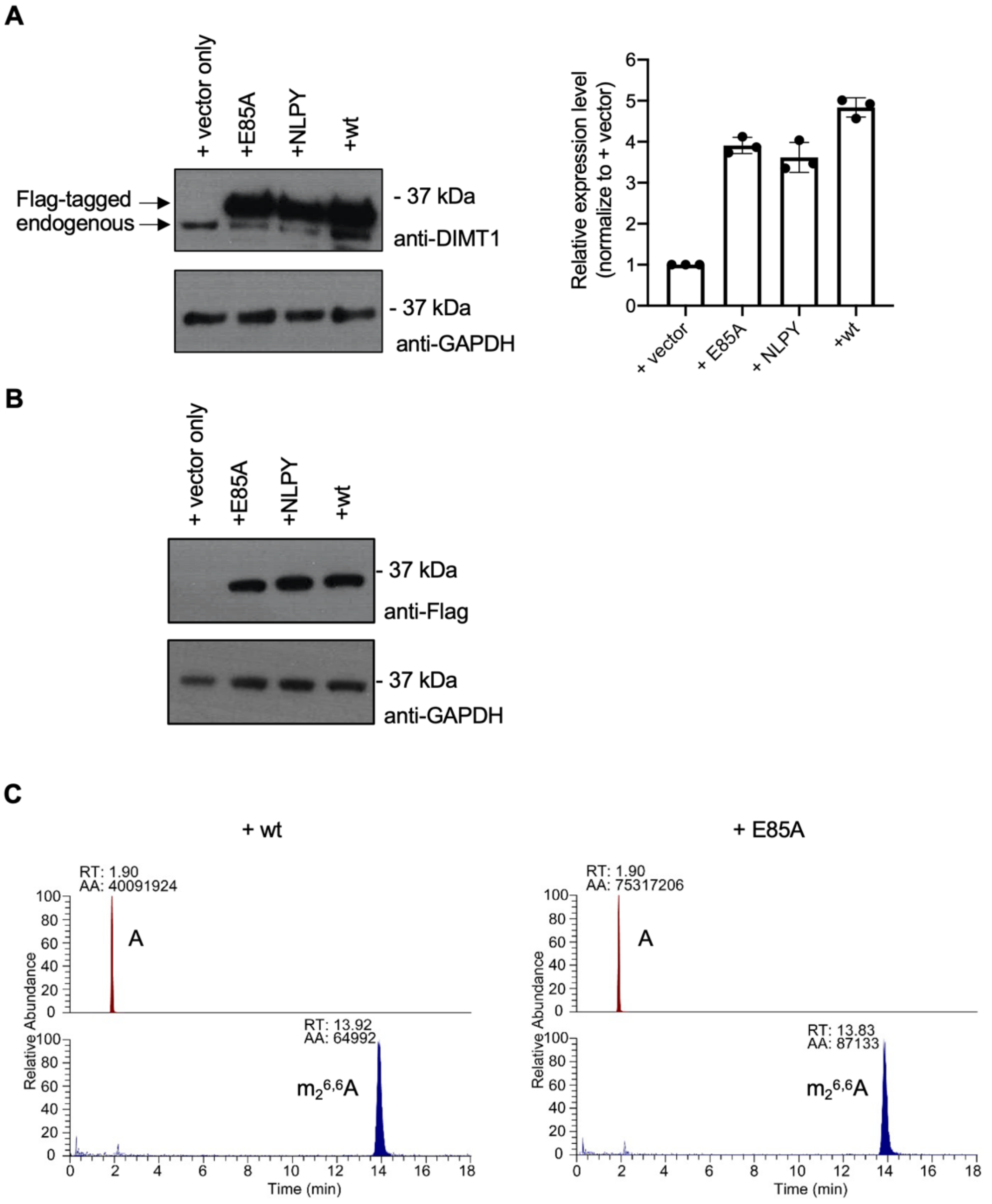

Figure S3. NLPY DIMT1 is catalytically inactive. (A) Left: Western blots showing the expression of DIMT1 (both Flag-tagged and endogenous) in *DIMT1*$^{+/-}$ + E85A, *DIMT1*$^{+/-}$ + wild type (wt), *DIMT1*$^{+/-}$ + NLPY (wt), and *DIMT1*$^{+/-}$ + empty vector cells with anti-DIMT1 antibody. Right: Quantification of the expression of DIMT1 in these cells. (B) Western blots showing the expression of FLAG-tagged DIMT1 in *DIMT1*$^{+/-}$ + E85A, *DIMT1*$^{+/-}$ + wild type (wt), *DIMT1*$^{+/-}$ + NLPY, and *DIMT1*$^{+/-}$ + empty vector with anti-Flag antibody. (C) LC-MS/MS channels and peak areas of adenosine and $m_2^{6,6}A$ in small RNA (< 40 nt) extracted from *DIMT1*$^{+/-}$ + wild type DIMT1 (left) and *DIMT1*$^{+/-}$ + E85A DIMT1.

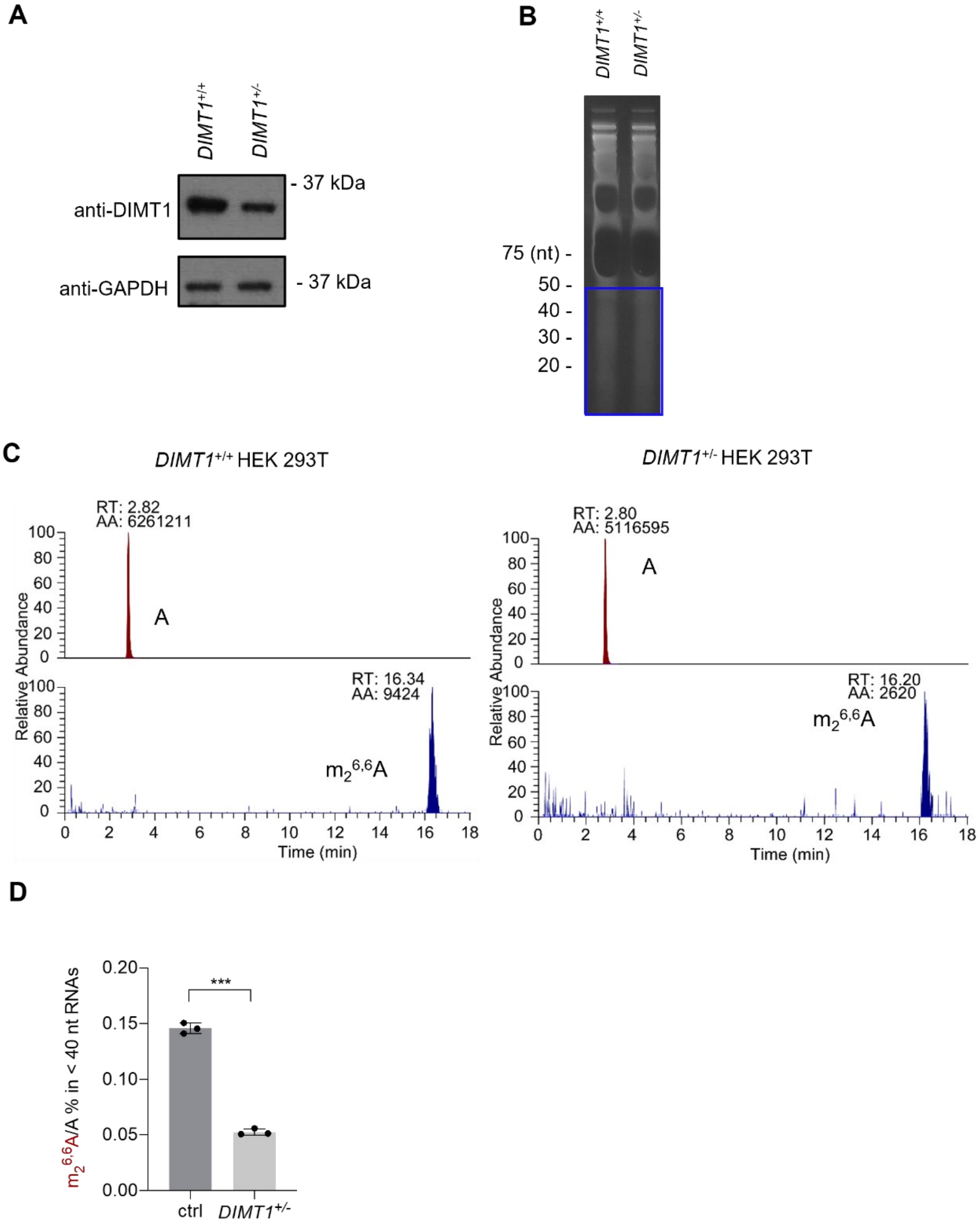

Figure S4. (A) Western blots showing the expression of endogenous DIMT1 in *DIMT1*$^{+/+}$ and *DIMT1*$^{+/-}$ HEK 293T cells. (B) Gel purification of small RNAs from *DIMT1*$^{+/+}$ and *DIMT1*$^{+/-}$ HEK 293T cells. (C) LC-MS/MS channels and peak areas of adenosine and $m_2^{6,6}A$ in small RNA (< 40 nt) extracted from *DIMT1*$^{+/+}$ (left) and *DIMT1*$^{+/-}$ (right) HEK 293T cells. (D) quantification of $m_2^{6,6}A$ in small RNA (< 40 nt) from *DIMT1*$^{+/+}$ (ctrl) and *DIMT1*$^{+/-}$ HEK 293T cells.

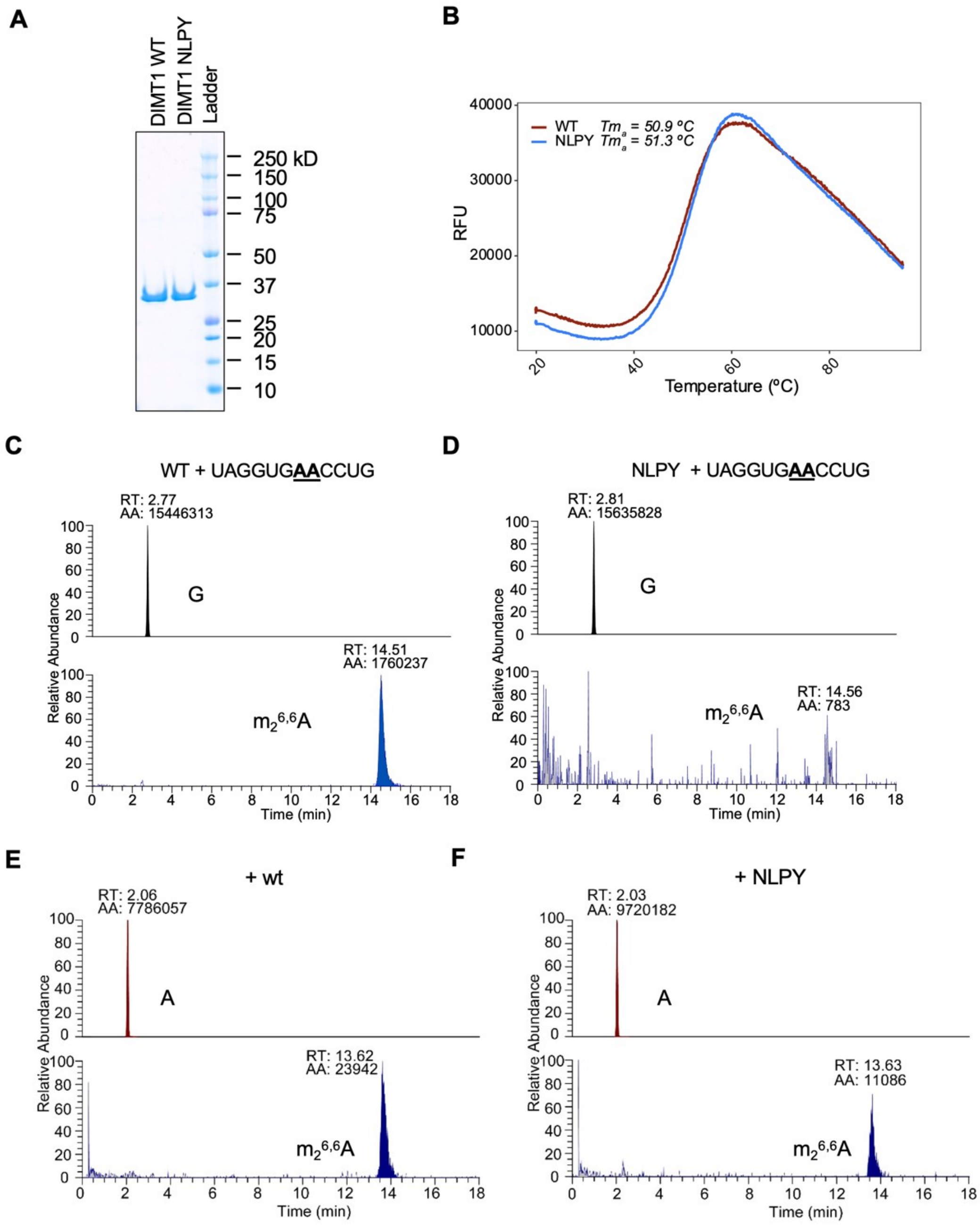

Figure S5. NLPY DIMT1 is catalytically inactive. (A) SDS-PAGE results of purified wild type and DIMT1 NLPY variant. (B) Thermostability of WT-DIMT1 and NLPY DIMT1 variant. RFU means relative fluorescence units. LC-MS/MS channels and peak areas of guanosine and $m_2^{6,6}A$ in the *in vitro* reactions with (C) wild type or (D) NLPY DIMT1 variant. LC-MS/MS channels and peak areas of adenosine and $m_2^{6,6}A$ in (E) *DIMT1*$^{+/-}$ + wild type (wt) or (F) *DIMT1*$^{+/-}$ + NLPY cells.

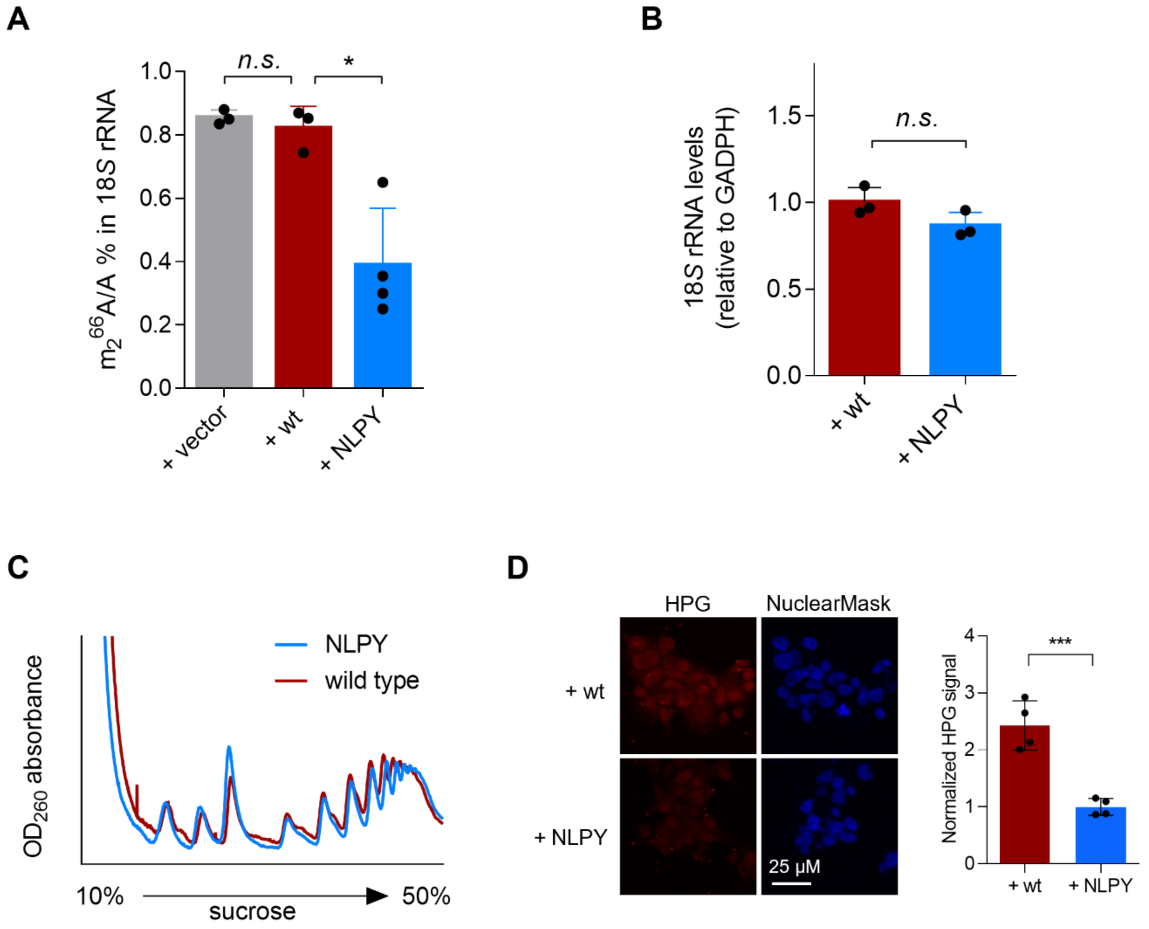

Figure S6. Expression of the catalytically inactive NLPY DIMT1 decreases $m_2^{6,6}$A in 18*S* rRNA and impair protein synthesis but does not lead to obvious changes in the levels of 18*S* rRNA or 40*S* monosome. (**A**) LC-MS/MS quantification of $m_2^{6,6}$A in 18*S* rRNA extracted from *DIMT1*$^{+/-}$ + empty vector, *DIMT1*+/- + wild type (wt), and *DIMT1*$^{+/-}$ + NLPY cells. *p* values were determined using a two-tailed Student's t-test for unpaired samples. Error bars represent mean ± SD. ***p < 0.001, and *n.s.* = p > 0.05. (**B**) qRT-qPCR quantification of 18*S* rRNA as normalized the level of GADPH in *DIMT1*$^{+/-}$ + wild-type DIMT1 and *DIMT1*$^{+/-}$ + NLPY DIMT1 cells. *p* values were determined using a two-tailed Student's t-test for unpaired samples. Error bars represent mean ± SD; *n.s.* = p > 0.05. (**C**) Polysome profiles of *DIMT1*$^{+/-}$ + wild type and *DIMT1*$^{+/-}$ + NLPY variant cells. (**D**) Imaging and quantification of fluorescent-labeled nascent protein in *DIMT1*$^{+/-}$ + wild type and *DIMT1*$^{+/-}$ + NLPY cells. Nuclei were stained with NuclearMask.

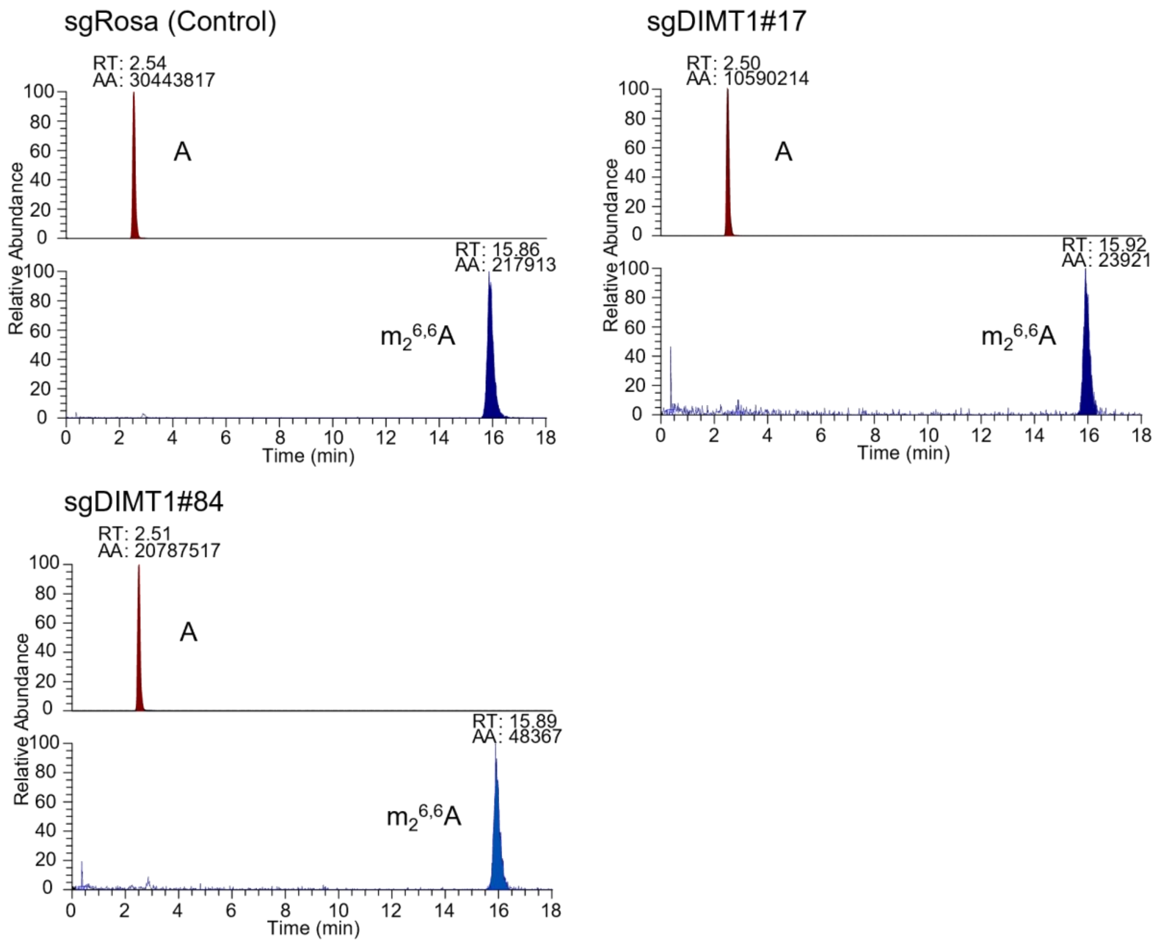

Figure S7. Downregulation of DIMT1 in MOLM-13C leads to a decreased level of $m_2^{6,6}$A in small RNAs. LC-MS/MS channels and peak areas of adenosine and $m_2^{6,6}$A MOLM-13 cells transfected with sgCtrl, sgDIMT1#17, or sgDIMT1#84.
